# RACK1 in host immune response to infections: molecular mechanisms and therapeutic potentials

**DOI:** 10.3389/fimmu.2026.1859544

**Published:** 2026-06-24

**Authors:** Zhenglin He, Yishuo Ji, Hanming Hao, Kai Zhao, Shangwei Ji, Liang Han, Yue Hu

**Affiliations:** 1 Department of Biobank, China-Japan Union Hospital of Jilin University, Jilin University, Changchun, Jilin, China; 2 Department of Infectious Diseases, China-Japan Union Hospital of Jilin University, Changchun, Jilin, China; 3 Department of Pathology, China-Japan Union Hospital of Jilin University, Changchun, Jilin, China; 4 Infectious Diseases and Pathogen Biology Center, State Key Laboratory for Diagnosis and Treatment of Severe Zoonotic Infectious Diseases, Key Laboratory for Zoonosis Research of the Ministry of Education, The First Hospital of Jilin University, Changchun, Jilin, China; 5 Department of Biomedical Science, College of Basic Medical Sciences, Jilin University, Changchun, Jilin, China; 6 Department of Immunobiology, Yale University School of Medicine, New Haven, CT, United States; 7 Center of Molecular and Cellular Oncology, Yale Cancer Center, Yale University, New Haven, CT, United States

**Keywords:** adaptive Immunity, host-pathogen interaction, innate immunity, RACK1, scaffolding protein, signal transduction, translational control

## Abstract

The global healthcare system faces increasing threats from emerging and re-emerging pathogens. Current understanding of host-pathogen interactions and underlying immune mechanisms remains incomplete, which hinders the development of effective diagnostic tools and therapeutic strategies. The receptor for activated C kinase 1 (RACK1) is a multifunctional scaffolding protein that integrates diverse signaling pathways, modulates translation, and regulates key cellular processes. Despite accumulating evidence implicating RACK1 in host immune response to infections, its multifaceted roles and mechanisms remain poorly defined. Hence, this review systematically discusses the involvement of RACK1 in host defense against bacterial and viral pathogens, with a focus on its regulation of inflammatory signaling, inflammasome activation, hormone-mediated immune regulation, reactive oxygen species production, adaptive immunity, and different pathogen infections. Together, current evidence suggests that RACK1 links signaling and translation to shape immune responses in different infectious settings and may provide a basis for host-directed therapeutic strategies.

## 1 Introduction

In the 21st century, the global healthcare system facing increased burden due to the emerging pathogen (1, 2). The global pandemic caused by severe acute respiratory syndrome coronavirus 2 (SARS-CoV-2), which began in December 2019, led to a surge in infections, which overwhelmed the existing medical resources and infrastructure (3). Our limited knowledge of virus-host interaction and host immune responses has further hampered the development of accurate and effective diagnostic and therapeutic strategies, both of which are essential for controlling the pandemic. Meanwhile, the situation of emerging antimicrobial resistance (AMR) pathogens is alarming. In 2019, bacterial AMR was directly responsible for 1.27 million deaths and contributed to about 4.95 million deaths globally. It is predicted that the global number of AMR related deaths will reach 8.2 million by 2050. In fact, the methicillin-resistant *Staphylococcus aureus* (MRSA) related deaths in 2021 was doubled compared to 1990 (2). The carbapenem resistant bacteria related deaths have increased from 619,000 in 1990 to 1.03 million in 2021 and the attributed deaths rose from 127,000 to 216,000 (2). Influenza viruses can be re-emerging and emerging pathogen, which constantly posting challenge on our healthcare system alongside other emerging pathogens (4). The latest data from WHO shows that seasonal influenza alone can cause a billion cases of infections each year, including an estimated 3–5 million severe cases and 290,000-650,000 deaths worldwide (5). Moreover, a new strain of influenza virus can emerge due to antigenic shift featured with dramatic reorganization of the virus genome leading to significant difference with the strains that the society has previously experienced and capable of causing pandemics (6). Thus, to be better prepared for the unpredictable emerging pathogens and reduce the impact of ongoing threats, researchers have invested significant effort into understanding host-pathogen interactions (7). Gaining a detailed understanding of host immune responses and pathogen immune-evasion mechanisms is essential for defining host-pathogen interactions and can guide the development of improved biomarkers, vaccines, host-directed therapies, and other therapeutic strategies (8–10).

**Figure 1:**
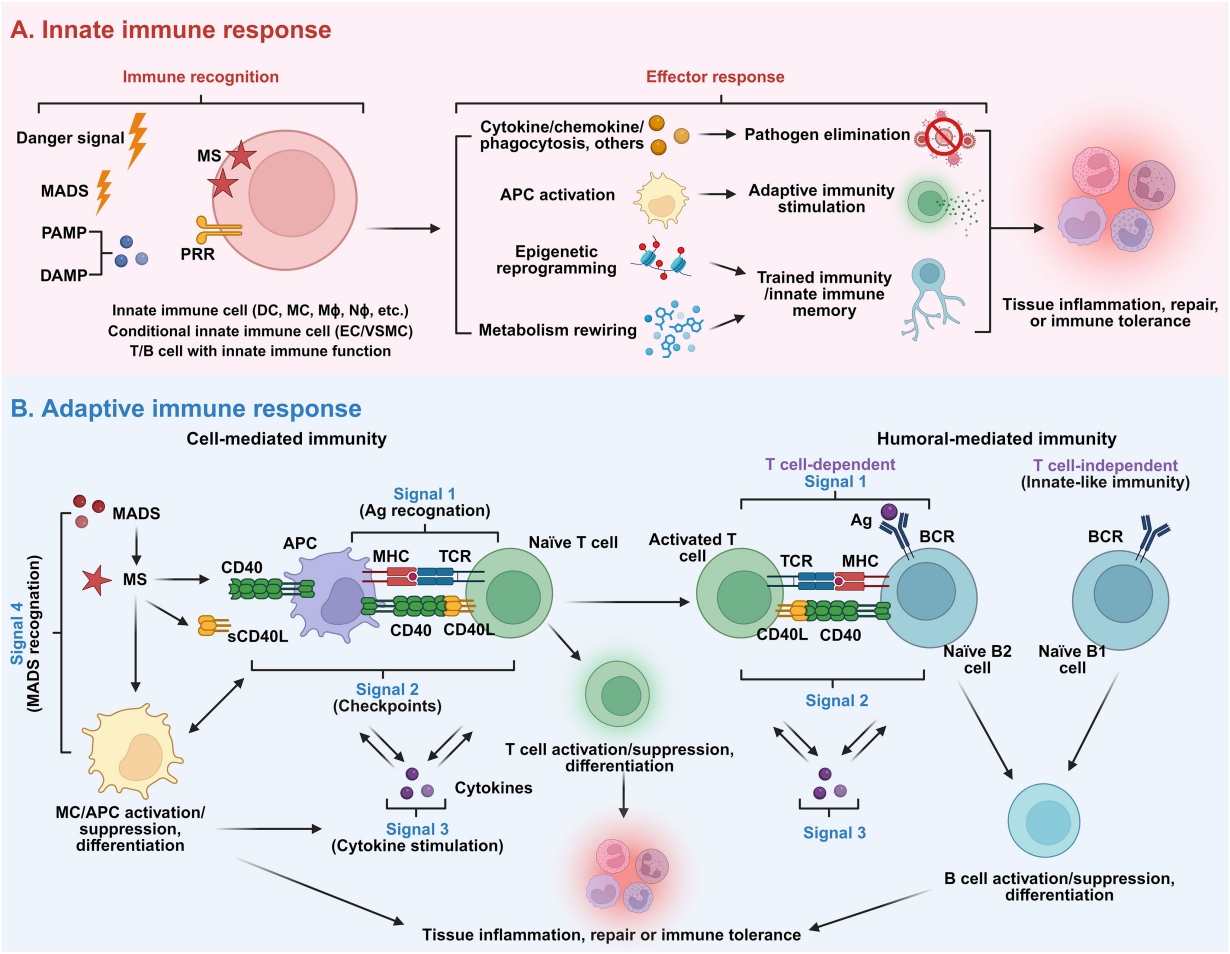
Innate-adaptive immunity interplay in immune response. **(A)** Innate immune response. Immune recognition (MADS: MS and PAMP/DAMP: PRR) initiates innate immune response leading to pathogen elimination, adaptive immunity stimulation, and trained immunity via cytokine, chemokine, phagocytosis response, APC activation, and epigenetic reprogramming/metabolism rewiring, and contributes to tissue inflammation or repair. **(B)** Adaptive immune response. Adaptive immunity determines tissue inflammation and repair, consisting of cell- and humoral-mediated immunity. Four signals are involved in cell-mediated immunity (Ag recognition, checkpoint, cytokine stimulation, and MADS recognition). CD40:CD40L is a representative immune checkpoint molecular pair. There are 2 types of humoral immune response (T cell-dependent and -independent). Three signals are described for T cell-dependent B cell immune response (Ag recognition, immune checkpoint and cytokine stimulation). B cell can also respond to Ag without the participation of Th cell in T cell-independent immunity. Ag, antigen; APC, antigen-presenting cell; BCR, B-cell receptor; CD40, cluster of differentiation 40; CD40L, CD40 ligand; DC, dendritic cell; EC, endothelial cell; MADS, metabolite-associated danger signal; MC, monocyte; Mφ, macrophage; MHC, major histocompatibility complex; MS, metabolic sensor; Nφ, neutrophil; PAMP, pathogen-associated molecular pattern; PRR, pattern recognition receptor; sCD40L, soluble CD40 ligand; TCR, T-cell receptor; VSMC, vascular smooth muscle cell.

The immune system protects the host through coordinated and integrated innate and adaptive immune responses that jointly interpret pathogenic signals and orchestrate context-appropriate effector programs (11, 12) (**Figure 1**). Innate immunity provides the first line of defense against invading pathogens. Infected cells, dendritic cells, and macrophages detect conserved microbial structures through pattern-recognition receptors (PRRs), which recognize pathogen-associated molecular patterns (PAMPs) and initiate innate immune signaling (13). Upon ligand recognition, PRRs activate intracellular signaling cascades, including nuclear factor kappa-light-chain-enhancer of activated B cells (NF-κB) and mitogen-activated protein kinase (MAPK) pathways, thereby inducing inflammatory cytokines, chemokines, interferons, and other antimicrobial mediators that promote local inflammation and recruit effector leukocytes (14–16). In parallel, antigen-presenting cells process microbial antigens and display peptide fragments in complex with major histocompatibility complex (MHC) molecules for T cell recognition, thereby providing a critical antigenic signal for adaptive immune activation (17, 18). Through these coordinated inflammatory and antigen-presenting programs, innate immune recognition links early pathogen sensing to the magnitude, quality, and duration of adaptive immune responses (19). The adaptive immune system subsequently mounts antigen-specific responses mediated by antibodies and specialized lymphocyte populations, and generates long-lived immunological memory that enables accelerated and amplified responses upon re-exposure (20, 21). Beyond pathogen clearance, immune processes also contribute to tissue and cellular homeostasis by coordinating inflammatory resolution, tissue repair, and metabolic adaptation in immune and tissue-resident cells (22, 23). In addition, interactions between the immune system and the commensal microbiota support resistance to infection by maintaining epithelial barrier function, shaping local immune tone, and providing colonization resistance against invading pathogens (24, 25).

The receptor for activated C kinase 1 (RACK1) was initially identified in 1991 as a protein kinase C (PKC) anchoring protein (26). Over decades of research, RACK1 has become widely recognized as a multifunctional scaffolding protein that interacts with a diverse set of proteins and complexes (27), including signaling proteins, kinases, phosphatases (28), ion channel proteins, transmembrane receptors (29), ribosomes, autophagy initiation complexes (30), centrosomes, inflammasomes (31), and others yet to be identified (32). It facilitates the assembly of signaling complexes by recruiting signaling proteins involved in various pathways such as cyclic adenosine monophosphate (cAMP), MAPK, Src family tyrosine kinase (Src), Wingless/Int-1 (Wnt) signaling pathway, and NF-κB (33). Through these interactions, RACK1 recruits its binding partners, such as PKCs, to their sites of action across different pathways, thereby promoting cross-talk between signaling cascades (34). In addition, RACK1 is a ribosome-associated scaffold protein localized on the 40S ribosomal subunit, where it serves as a docking platform for signaling and translational regulators, thereby contributing to translation initiation and selective mRNA translation (29, 35). Furthermore, RACK1 can affect the stability of its binding partners by either targeting them for degradation or protecting them from it, such as proteins involved in apoptosis and oxidative stress (36). As a result, RACK1 has been reported to play regulatory roles in various fundamental cellular processes including cell growth, proliferation, and migration (37, 38), as well as in key cellular functions such as protein synthesis, protein degradation, apoptosis, and stress response (39).

RACK1 does not operate through a single immune pathway. Instead, it functions as a versatile scaffold that coordinates multiple cellular processes directly relevant to host defense and host-pathogen interactions (29, 40). In innate immunity, RACK1 serves as a signaling platform that amplifies inflammatory responses and cytokine production upon challenge by both viral and bacterial pathogens (41, 42). Concurrently, RACK1 governs adaptive immune competence by promoting B cell and T cell development, maturation, and effector differentiation (43–45). Notably, these RACK1-regulated processes are frequently exploited by pathogens to subvert host defenses and facilitate immune evasion. Together, these findings underscore RACK1’s critical role in regulating both innate and adaptive immunity and suggest that a comprehensive understanding of RACK1-dependent signaling circuits may be essential for the rational development of therapeutic strategies aimed at modulating host immune responses during infection.

Recent review summarizes RACK1 as a multifunctional scaffold and ribosomal regulator with context-dependent roles in pan-cancer signaling and therapeutic targeting (46). Despite increasing recognition of RACK1 in immune regulation, its precise roles and mechanisms in shaping host immune responses during infection remain poorly defined. To bridge this knowledge gap, this review will comprehensively discuss RACK1’s involvement in host defense against a broad spectrum of pathogens such as bacteria and viruses. We will discuss RACK1’s involvement in inflammatory signaling, regulation of autophagy, and control of cytoskeletal dynamics, which are crucial for pathogen clearance and the establishment of immune memory. Additionally, we will evaluate the potential of RACK1 as a therapeutic target in combating infectious diseases, and addressing the challenges posed by targeting such a multifaceted protein.

## 2 RACK1: structure and function

**Figure 2:**
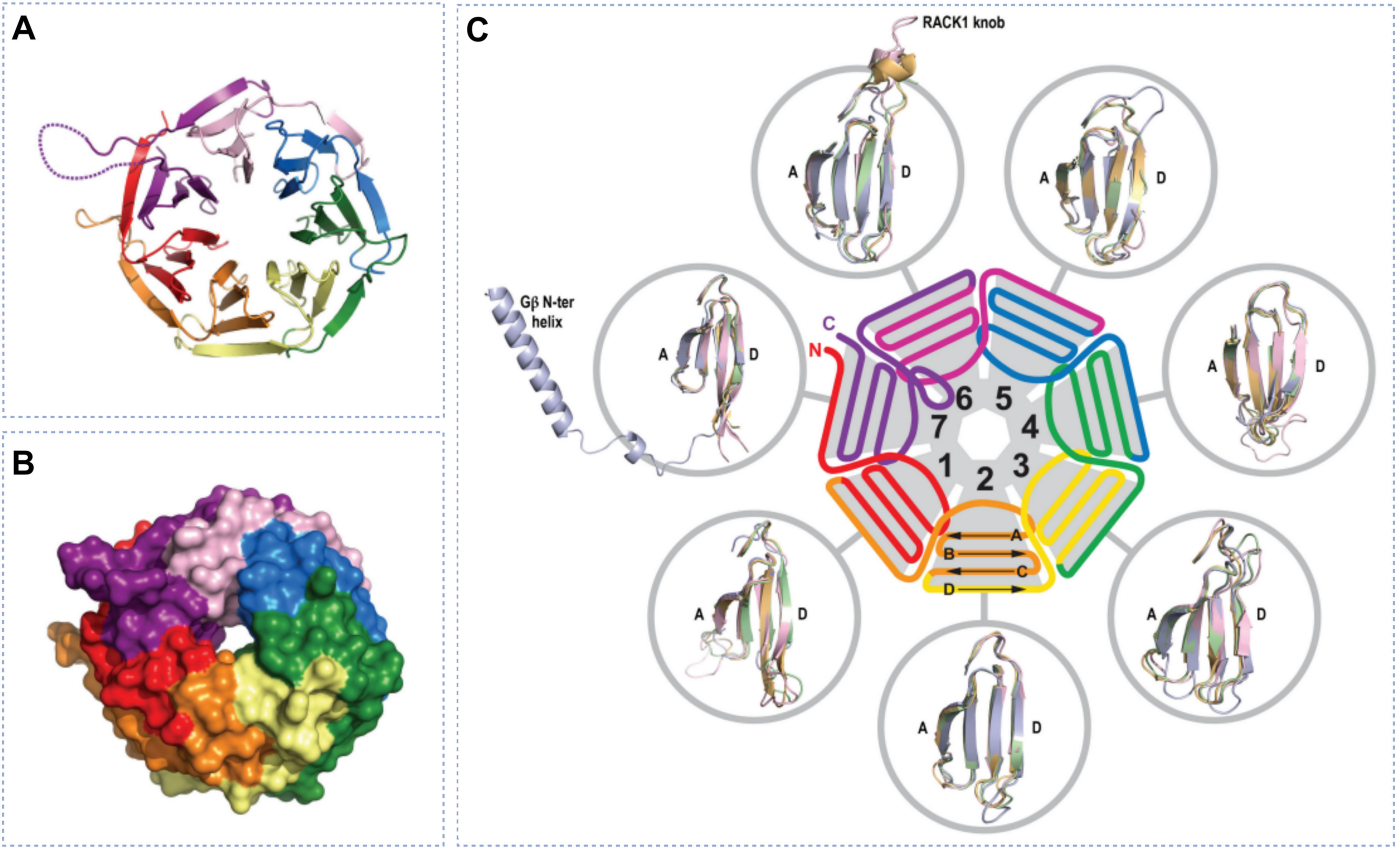
Structural detail for RACK1 proteins. **(A)** Crystal structure of RACK1A from A. thaliana (PDB: 3DM0), illustrating the seven-bladed β propeller structure. **(B)** As **(A)** but with surface rendition. **(C)** Schematic representation of RACK1 structure and organization of WD-repeats; peripheral circles show superimposition of individual propeller blades for Gb1 (blue) (PDB: 1TBG) and for the structurally defined RACK1 orthologues from A. thaliana (green) (PDB: 3DM0), S. cerevisiae (orange) (PDB: 3FRX) and (T) thermophila (pink) (PDB: 2XZN). Gb is distinguished in blade 7 by a helical N-terminal extension that engages tightly bound Gg (not shown) in coiled coil interactions. Reproduced from Adams et al. (47).

RACK1 is a highly conserved member of the WD40 repeat protein family, characterized by seven WD repeats that fold into a seven-bladed β-propeller architecture (32) (**Figure 2**). This compact propeller, closed by a canonical “velcro” motif between its N- and C-termini, forms a cylindrical structure approximately 50 Å in diameter with a central channel (~7 Å) and distinct electrostatic surfaces—an alkaline top and an acidic bottom—while prominently exposed surface loops on the top face provide versatile interfaces for protein–protein interactions (47, 48).

Each WD repeat contributes a four-stranded antiparallel β-sheet, with conserved tryptophan side chains intercalated into hydrophobic pockets to stabilize the propeller (48). Subtle variations in inter-blade loops and non-conserved residues (e.g., substitutions in the GH dipeptide motif) underlie species-specific conformational flexibility. The human RACK1 crystal structure, resolved at 2.45 Å (space group P4_1_2_1_2), reveals that it anchors as an intrinsic component of the 40S ribosomal subunit, positioned on the solvent-exposed backside of the head region and embedded within a conserved scaffold formed by ribosomal proteins uS3, uS9, eS17, and helices 39–40 of 18S rRNA (29). Extensive hydrogen-bonding, salt-bridge, and hydrophobic interactions (~1,800 Å² interface) secure this association, situating RACK1 adjacent to the mRNA entry and exit channels (49). Portions of blades 1, 2, and 4 are buried in ribosomal contacts, while exposed regions on blades 3, 5–7 provide versatile docking platforms for signaling and translational regulators such as PKC, Src, eIF4E, PDE4D5, and β-integrins (50–53). Post-translational modifications further modulate its scaffolding function: phosphorylation at Ser146 may promote dimerization via the interface between blades 3–4, and MARylation at Asp144, Glu145, and Asp203—highly accessible residues on blades 4–5—dynamically regulates interactions with partners including G3BP1 and eIF3η without disrupting ribosome association (54). These structural and regulatory features collectively position RACK1 at the interface of the ribosomal machinery and cellular signaling networks.

RACK1 was originally identified as a receptor for activated PKC, functioning as an anchoring platform that spatially constrains kinase activity within the cell (27). This early characterization established RACK1 as a molecular scaffold rather than a signaling enzyme, setting the conceptual framework for its subsequent functional expansion. Building on this role, RACK1 was later shown to assemble heterogeneous signaling complexes by simultaneously engaging kinases, phosphatases, and adaptor proteins, thereby modulating signal propagation and termination across multiple pathways (55). Depending on its interacting partners and cellular context, RACK1 can either potentiate or dampen downstream signaling outputs, influencing pathways such as MAPK, c-Jun N-terminal kinase (JNK), and NF-κB signaling (56).

A major conceptual advance emerged with the discovery that RACK1 is constitutively associated with the ribosome, revealing an unexpected link between signal transduction and the translational machinery (35). Through this ribosome engagement, RACK1 recruits regulatory factors, including kinases and RNA-binding proteins, to control translation initiation, ribosome quality surveillance, and the selective translation of specific mRNA cohorts (57). This ribosome-linked scaffolding positions RACK1 as a conduit through which extracellular and intracellular signals are directly integrated into protein synthesis programs, aligning translational output with growth conditions and cellular stress states (27, 58).

More recent work has further refined this model by demonstrating that post-translational modifications of ribosome-associated RACK1 dynamically tune its translational functions. These modifications regulate stress-induced translational reprogramming and stress granule dynamics, providing mechanistic insight into how environmental cues rewire protein synthesis at the ribosomal interface (27, 59). Beyond translation control, RACK1 continues to emerge as a modular adaptor within specialized signaling assemblies, including those involved in interferon-induced signal transducer and activator of transcription (STAT) activation and broader cytokine-responsive complexes (60). In parallel, RACK1 has been implicated in the regulation of cytoskeletal organization and calcium signaling (61), highlighting its capacity to coordinate biochemical signaling with structural and biophysical cellular processes.Through this intricate β-propeller framework, RACK1 acts as a structural and signaling hub on the ribosome, integrating translational control with diverse cellular pathways such as stress response, adhesion, and migration (57).

**Figure 3:**
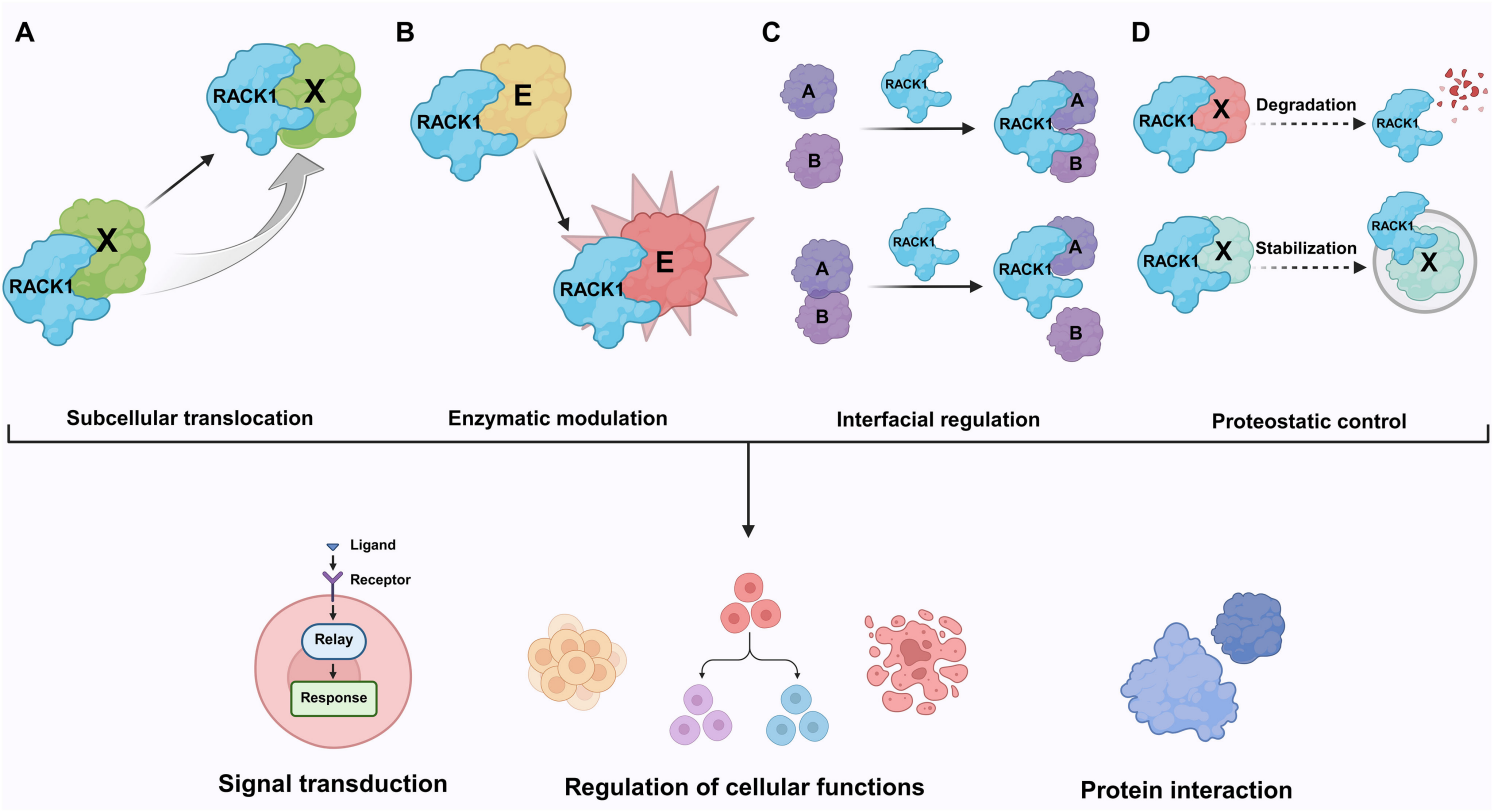
Different regulatory modes of RACK1 on its binding partners. **(A)** Cellular translocation of binding partners. **(B)** Enzymatic modulation via enzymatic activation. **(C)** Interfacial regulation on partner interactions. **(D)** Proteostatic control governing partner stability or degradation.

The versatility of RACK1’s seven-bladed β-propeller framework enables physical interactions with a diverse signaling proteome—encompassing kinases, phosphatases, ion channels, membrane receptors, and ribosomal components—that can be taxonomically divided into two spatial categories: soluble signaling proteins of cytoplasmic and nuclear compartments, and the cytosolic domains of membrane-spanning receptors (62). Functionally, these interactions manifest through four distinct molecular mechanisms: subcellular translocation (shuttling partners between compartments), enzymatic modulation (altering catalytic activity), interfacial regulation (enhancing or disrupting protein-protein associations), and proteostatic control (stabilizing or targeting partners for degradation) (**Figure 3**) (62). This mechanistic framework underlies the specific functional roles discussed above. These diverse regulatory capacities position RACK1 as a central molecular scaffold in immune cell signaling and host-pathogen interactions (60, 63).

## 3 Roles of RACK1 in host immune responses

**Figure 4:**
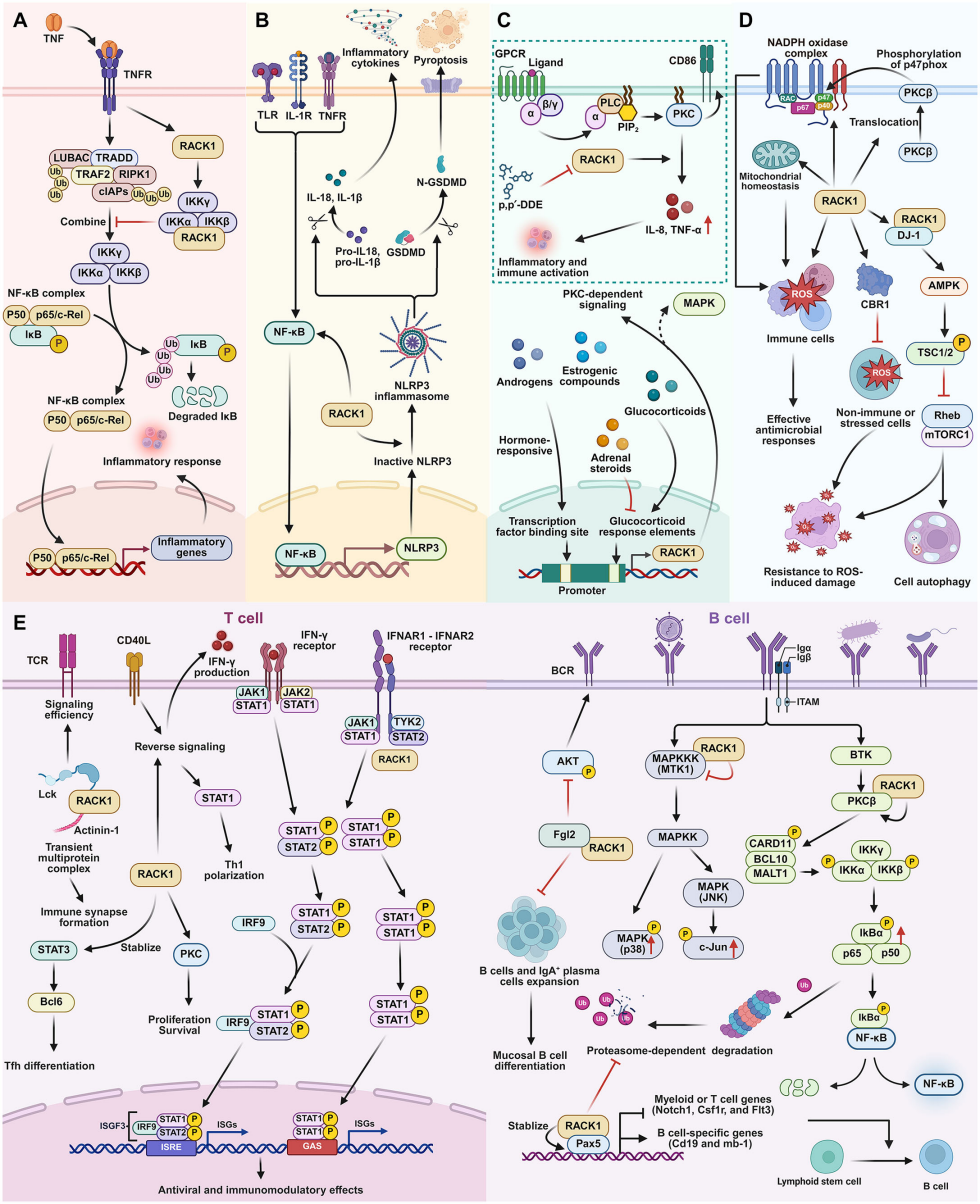
RACK1 exerts different effects in host immune response. **(A)** Inflammatory signaling pathways. RACK1 inhibits the binding of the IκB kinase (IKK) complex to the TNF receptor activation complex by interacting with IKKα and IKKβ, thereby suppressing the NF-κB pathway. **(B)** Inflammasome activation. RACK1 activates the NF-κB pathway and promotes the transcription of NLRP3. RACK1 is also involved in the assembly of NLRP3 inflammasome, thereby facilitating the production of IL-18, IL-1β, and N-GSDMD, leading to inflammatory cytokine generation and pyroptosis. **(C)** Hormone-mediated immune regulation. Glucocorticoids repress RACK1 through glucocorticoid response elements, while adrenal steroids reverse this effect. Estrogenic compounds and androgens enhance RACK1 transcription, promoting PKC-dependent signaling and MAPK pathway activity. RACK1 promotes PKCβ-dependent signaling and pro-inflammatory cytokine production, leading to inflammatory and immune activation. **(D)** ROS regulation. RACK1 plays a dual role in regulating ROS. In immune cells, it promotes antimicrobial responses by facilitating PKCβ-mediated assembly of the NADPH oxidase complex, enhancing superoxide production. Conversely, in non-immune or stressed cells, RACK1 suppresses excessive ROS accumulation to prevent oxidative damage. By stabilizing carbonyl reductase 1 (CBR1) to reduce ROS-induced injury and by activating the DJ-1/AMPK/mTOR pathway, RACK1 enhances cell stress resistance. **(E)** Adaptive immunity. T cell: Upon TCR stimulation, RACK1 interacts with Lck and actinin-1 to facilitate immune synapse formation and signaling efficiency. It promotes T follicular helper (Tfh) differentiation by stabilizing STAT3 and inducing Bcl6. B cell: RACK1 maintains B cell identity and development by binding Pax5, inhibiting its ubiquitination and proteasomal degradation. In mature B cells, RACK1 enables BCR-driven MAPK and NF-κB signaling. Additionally, RACK can suppress mucosal B cell differentiation by inhibiting AKT phosphorylation, and inhibit B cells and IgA^+^ plasma cells expansion. AKT, v-akt murine thymoma viral oncogene homolog (protein kinase B); AMPK, AMP-activated protein kinase; BCL10, B-cell lymphoma/leukemia 10; Bcl6, B-cell lymphoma 6 protein; BCR, B-cell receptor; BTK, Bruton’s tyrosine kinase; CARD11, caspase recruitment domain family member 11; CBR1, carbonyl reductase 1; CD40L, CD40 ligand; CD86, cluster of differentiation 86; cIAPs, cellular inhibitor of apoptosis proteins; c-Jun, cellular Jun (proto-oncogene); c-Rel, v-rel avian reticuloendotheliosis viral oncogene homolog; DDE, dichlorodiphenyldichloroethylene; DJ-1, Parkinson disease protein 7 (PARK7); GAS, gamma-activated sequence; GPCR, G protein-coupled receptor; GSDMD, gasdermin D; IFNAR1, interferon-alpha receptor 1; IFN-γ, interferon-gamma; Igα, immunoglobulin alpha; Igβ, immunoglobulin beta; IκB, inhibitor of nuclear factor kappa B; IKKα, inhibitor of nuclear factor kappa-B kinase subunit alpha; IL-1β, interleukin-1 beta; IL-1R, interleukin-1 receptor; IRF9, interferon regulatory factor 9; ISGF3, interferon-stimulated gene factor 3; ISGs, interferon-stimulated genes; ISRE, interferon-stimulated response element; ITAM, immunoreceptor tyrosine-based activation motif; JAK1, Janus kinase 1; JNK, c-Jun N-terminal kinase; Lck, lymphocyte-specific protein tyrosine kinase; LUBAC, linear ubiquitin chain assembly complex; MALT1, mucosa-associated lymphoid tissue lymphoma translocation protein 1; MAPK, mitogen-activated protein kinase; MAPKK, mitogen-activated protein kinase kinase; MAPKKK, mitogen-activated protein kinase kinase kinase; mTORC1, mechanistic target of rapamycin complex 1; MTK1, MAP three kinase 1; NADPH, reduced nicotinamide adenine dinucleotide phosphate; NF-κB, nuclear factor kappa-light-chain-enhancer of activated B cells; NLRP3, NLR family pyrin domain containing 3; N-GSDMD, N-terminal gasdermin D; p38, p38 mitogen-activated protein kinase; p50, nuclear factor kappa B subunit 1 (NF-κB p50); p65, transcription factor p65 (RelA); P, phosphorylation; Pax5, paired box 5; PIP2, phosphatidylinositol 4,5-bisphosphate; PKC, protein kinase C; PKCβ, protein kinase C beta; PLC, phospholipase C; p,p’-DDE, p,p’-dichlorodiphenyldichloroethylene; RACK1, receptor for activated C kinase 1; Rheb, Ras homolog enriched in brain; RIPK1, receptor-interacting protein kinase 1; ROS, reactive oxygen species; STAT1, signal transducer and activator of transcription 1; TCR, T-cell receptor; Tfh, T follicular helper cell; Th1, T helper 1 cell; TLR, Toll-like receptor; TNF, tumor necrosis factor; TNF-α, tumor necrosis factor-alpha; TNFR, tumor necrosis factor receptor; TRADD, TNF receptor-associated death domain protein; TRAF2, TNF receptor-associated factor 2; TSC1/2, tuberous sclerosis complex 1/2; Ub, ubiquitin.

RACK1 is a multifunctional scaffolding protein that orchestrates a variety of cellular signaling pathways, significantly shaping host immune responses across species (43, 60). Its roles have been implicated in regulation of inflammatory signaling cascades, inflammasome assembly, hormonal regulation of immune signaling, reactive oxygen species (ROS) production, and adaptive immunity (42, 43) (**Figure 4**).

### 3.1 RACK1’s role in inflammatory signaling pathways

Inflammatory signaling pathways play a crucial role in regulating host immune responses, particularly in the context of pathogen recognition and immune activation. NF-κB represent a family of inducible transcription factors function as the master mediator of the expression of pro-inflammatory genes in both innate and adaptive immune cells (64). Under physiological conditions, NF-κB activity is tightly restrained, whereas inflammatory or stress signals rapidly activate the canonical NF-κB pathway by converging on the IκB kinase (IKK) complex, which functions as the central regulatory node of the pathway (65). The IKK complex, consisting of the catalytic subunits IKKα and IKKβ and the regulatory subunit NEMO (IKKγ), phosphorylates inhibitory IκB proteins, targeting them for ubiquitin-dependent degradation and thereby permitting NF-κB dimers to translocate into the nucleus and initiate gene transcription (66). The NF-κB pathway can be activated through the recognition of PAMPs by PRRs, as well as by the ligand binding of the TNF receptors, cytokine receptors, T-cell receptors and B-cell receptors (67).

Under unstimulated conditions, RACK1 functions as a negative regulator of the NF-κB signaling pathway. Mechanistically, RACK1 exerts its inhibitory effect through direct protein–protein interactions, which represent the most characteristic feature of this scaffold protein (29). Specifically, RACK1 physically associates with the IKK complex, preventing its recruitment to TRAF2—a key adaptor molecule within the TNF receptor activation complex (33). This blockade interrupts the downstream signaling cascade that normally leads to phosphorylation and degradation of IκBα, release of NF-κB dimers p65/p50, and their subsequent translocation into the nucleus to initiate transcription of pro-inflammatory target genes (67). Consequently, RACK1-mediated interference results in the inhibition of NF-κB nuclear translocation and transcriptional activation. Notably, although this mechanism has been primarily characterized in the context of TNF-induced NF-κB activation, whether RACK1 exerts similar inhibitory effects on NF-κB signaling triggered by other receptor pathways (such as IL-1R) remains to be fully elucidated (33).

Loss of this RACK1-dependent restraint shifts NF-κB signaling toward a lower activation threshold, a change that becomes particularly evident under conditions of persistent microbial exposure and ongoing inflammatory stress. Diminished RACK1 expression is associated with exaggerated NF-κB activity in *Helicobacter pylori* (*H. pylori*)-driven gastritis (68). *H. pylori* infection suppresses RACK1 expression, leading to the upregulation of integrin β1 and subsequent activation of the NF-κB signaling pathway (68). While this pathway is a critical component of host defense, mediating proinflammatory cytokine production to eliminate pathogens, in the context of *H. pylori* infection such activation becomes maladaptive (69). The downregulation of RACK1 appears to favor bacterial persistence, as increased integrin β1 expression on the gastric epithelial surface facilitates *H. pylori* adhesion and colonization (68, 70). Meanwhile, NF-κB–driven chronic inflammation contributes to tissue damage and promotes the progression from persistent gastritis to gastric carcinogenesis (71). Thus, RACK1 downregulation not only reflects a bacterial strategy to evade immune clearance but also establishes a microenvironment conducive to *H. pylori* survival and tumorigenic transformation (72).

During classical swine fever virus (CSFV) infection, porcine RACK1 exerts a complex regulatory role in NF-κB signaling. Knockdown of RACK1 enhances viral replication but simultaneously leads to increased NF-κB activation, indicating that RACK1 modulates both antiviral and proinflammatory responses through distinct mechanisms (73). This finding suggests that RACK1 fine-tunes NF-κB signaling to balance inflammatory control and antiviral immunity. Disruption of RACK1 uncouples these processes, leading to exaggerated NF-κB activation but impaired antiviral restriction, thereby favoring CSFV replication (73).

In contrast, during *Pasteurella multocida* (*P. multocida*) infection, RACK1 is indispensable for effective NF-κB signaling (42). In this context, Ran et al. showed that depletion of RACK1 in macrophages attenuates *P. multocida*-induced NF-κB activation, as reflected by reduced p65 abundance and phosphorylation, together with a broad suppression of NF-κB-dependent pro-inflammatory cytokine (IL-6, IL-12, and TNF-α) and chemokine (CXCL1 and CXCL2) expression. This study identifies RACK1 as a positive regulator of NF-κB-driven inflammatory responses in *P. multocida* infection, highlighting a context-dependent role for RACK1 in antibacterial immunity (42).

Collectively, these findings suggest that RACK1 differentially modulates NF-κB signaling in a context-dependent manner, with its regulatory outcome varying according to the type of pathogen and the cellular environment.

### 3.2 RACK1’s role in inflammasome activation

Inflammasomes are cytosolic innate immune signaling platforms that sense microbial invasion and cellular stress, coupling danger recognition to inflammatory cytokine production and lytic cell death, thereby shaping early host defense and inflammatory outcomes (74).

The NLRP3 inflammasome is a cytosolic multiprotein complex of the innate immune system that is activated in a two-step manner, involving NF-κB–dependent priming and subsequent assembly in response to a wide range of pathogen- and danger-associated signals (75, 76). Upon activation, NLRP3 promotes caspase-1–mediated maturation of the pro-inflammatory cytokines IL-1β and IL-18 and triggers pyroptotic cell death, thereby playing a central role in host defense by amplifying inflammatory responses and restricting pathogen replication (77).

The NLRP3 inflammasome is composed of the sensor NLRP3, the adaptor ASC, and the effector caspase-1, whose assembly critically depends on the serine/threonine kinase NEK7 as a structural licensing factor (78). In this regard, RACK1 was first identified as a scaffolding protein that facilitates NLRP3 inflammasome assembly and activation by directly interacting with both NLRP3 and NEK7 in mouse macrophages stimulated with LPS and ATP (31). Recent work has further revealed that *Mycobacterium tuberculosis* (*M. tuberculosis*) secretes the effector protein EST12, which binds to RACK1 to form a complex that recruits the deubiquitinase UCHL5, removing K48-linked ubiquitin chains from NLRP3, thereby stabilizing and activating the complex. The resulting cascade activates caspase-1, induces GSDMD cleavage, and triggers IL-1β secretion and pyroptotic cell death, collectively restricting *M. tuberculosis* replication and reducing bacterial load in infected macrophages (40).

In *P. multocida* infection, RACK1 similarly promotes the assembly of the NLRP3–ASC–caspase-1 inflammasome complex, but it also modulates inflammasome-related gene expression through the NF-κB pathway. Specifically, RACK1 knockdown suppresses NF-κB activation and significantly decreases the transcription of IL-1β, IL-6, IL-12, and TNF-α, resulting in attenuated inflammatory responses in infected macrophages (42). The finding highlights RACK1 as a dual regulator that coordinates both inflammasome activation and transcriptional priming during bacterial infection.

The MAPK pathway plays a crucial role in regulating inflammasome activation and immune responses, particularly through its interaction with key signaling molecules. This pathway consists of three core kinase cascades-p38, JNK, and ERK-that transmit pathogen-induced signals downstream of PRRs in innate immune cells (79, 80). By regulating transcription factors such as AP-1 and NF-κB, MAPK signaling shapes inflammatory gene expression rather than cytokine processing itself (81). In studies using *Caenorhabditis elegans* (*C. elegans*) as the model organism, RACK1 has emerged as a conserved integrator of innate immune signaling, particularly through its engagement with MAPK pathways (82). It engages the MAPK axis at the level of signal propagation, positioning it upstream of inflammasome effector mechanisms (83). This organization provides a framework in which RACK1 influences inflammasome-related outcomes indirectly through modulation of inflammatory signaling process. RACK1 is required for effective defense against *Shigella flexneri* (*S. flexneri*) infection, acting upstream or in coordination with conserved MAPK signaling to promote host resistance (82, 84). These findings establish RACK1 as a key modulator of antimicrobial immunity via evolutionarily conserved kinase cascades rather than as a direct effector molecule.

Consistent with this role, studies in mammalian macrophages infected with *P. multocida* indicate that RACK1 primarily enhances NF-κB-dependent transcriptional priming of inflammatory genes, while its involvement in MAPK signaling appears indirect or context-dependent (42). Although inflammasome-related genes are among the transcriptional targets affected, current evidence supports a model in which RACK1 functions mainly at the level of upstream signaling coordination, rather than as a core component of inflammasome assembly or activation per se (31, 42).

In a *Klebsiella pneumoniae* infection model in *C. elegans*, *Lactobacillus casei* treatment activated RACK1 through a TLR-mediated p38 MAPK pathway (85, 86). Activated RACK1 subsequently amplifies antimicrobial gene expression and host defense responses, underscoring its capacity to couple MAPK signaling to pathogen-specific immune outputs (86). Existing evidence instead supports a view in which RACK1 operates primarily as an upstream signaling coordinator, coupling MAPK pathway activation to innate immune transcriptional outputs.

In this setting, RACK1 functions as a scaffold that facilitates signal integration rather than serving as a structural element of inflammasome complexes. Although transcriptional changes in inflammasome-associated genes are observed following RACK1 perturbation, these effects appear to arise from altered signaling dynamics and pathway crosstalk, with any involvement in inflammasome activation remaining indirect and dependent on cellular and inflammatory context.

### 3.3 RACK1’s role in hormone-mediated immune regulation

There is growing evidence that hormones play a role in mediating immune regulation, and they are widely used clinically to manage infection-associated inflammation. Glucocorticoids, the most potent class of anti-inflammatory drugs, play a pivotal role in the treatment of various immune-mediated inflammatory conditions. By modulating immune responses primarily through transcriptional control of inflammatory gene networks, glucocorticoids suppress pathological inflammation while broadly constraining immune cell activation and effector function (87, 88). These properties have led to their use in stabilizing immune responses in conditions such as bacterial sepsis (89). Dexamethasone, a corticosteroid drug with exclusively glucocorticoids activity, has been shown to attenuate inflammatory cytokine release via Kv1.3 suppression, contributing to its immunosuppression function in treating severe COVID-19 (90, 91).

Beyond their classical anti-inflammatory effects, steroid hormones also participate in immune regulation by reshaping intracellular signaling competence through transcriptional control of scaffold proteins and signaling adaptors, thereby linking endocrine status to immune responsiveness (92). Hormonal regulation of immune signaling is increasingly understood as a balance between glucocorticoids and adrenal androgens, which together determine cellular sensitivity to inflammatory stimuli and cytokine production capacity (92). Recent studies show that RACK1 contains specific hormone-responsive transcription factor binding sites in its promoter region that allow regulation by androgens and glucocorticoids. RACK1 expression is also modulated by androgens and glucocorticoids through specific transcription factor binding sites within its promoter (93). Notably, the RACK1 promoter harbors glucocorticoid response elements that mediate transcriptional repression by cortisol, whereas adrenal steroids such as dehydroepiandrosterone (DHEA) counteract this effect and restore RACK1 expression and downstream signaling activity (92). These hormonal effects are therefore poised to influence PKC-dependent signaling and may indirectly modulate MAPK pathway activity (94). Estrogenic compounds 17β-estradiol, diethylstilbestrol, and zearalenone were found to enhanced the transcription of RACK1 and RACK1/PKCβ-dependent signaling in THP-1 cells, leading to increased LPS-induced IL-8, TNF-α production, and CD86 expression (95). The endocrine-disrupting chemical p,p′-DDE downregulated RACK1 levels and suppressed LPS-induced immune activation (95, 96). In THP-1 cells, LPS stimulation induces robust TNF-α and IL-8 expression, as well as upregulation of the surface marker CD86, all of which are significantly modulated by changes in RACK1 levels under hormonal or xenobiotic influence (96). Thus, the use of steroids may indirectly reshape host-pathogen interactions by altering RACK1-mediated immune responses during infection.

Moreover, RACK1’s immunomodulatory capacity extends beyond classical pathogen sensing to broader host defense mechanisms (44). Experimental evidence indicates that RACK1 shapes immune responses in a cell-type–dependent manner during infection. In macrophages, RACK1 affects cytokine production through its influence on inflammasome-associated signaling following bacterial stimulation. In contrast, studies in lymphocytes point to a role for RACK1 in antigen-driven signaling cascades that guide activation and effector differentiation (42, 97). Transcriptomic and proteomic profiling in multiple infection models further indicate that RACK1 expression correlates with key innate and adaptive immune pathways, including Toll-like receptor signaling, p38 MAPK activation, and NF-κB-dependent transcription of proinflammatory mediators (43, 63, 98). Given that steroid hormones regulate RACK1 transcription and signaling activity, physiological changes in hormonal balance, such as alterations in the cortisol to DHEA ratio during stress or aging, may directly affect immune responses and inflammatory outcomes (92). Furthermore, given that sex hormones modulate RACK1 expression and immune signaling, physiological fluctuations in hormonal status may further influence RACK1-dependent immune regulation, with potential implications for infection susceptibility and outcome (96).

Taken together, RACK1 serves as a key integrator of hormonal and immune signals. Its sensitivity to both endogenous hormones and hormone-mimicking chemicals underscores its potential as a biomarker and therapeutic target in disorders of endocrine–immune interaction.

### 3.4 RACK1-mediated regulation of ROS

ROS are oxygen-derived metabolites, including superoxide, hydrogen peroxide, and hydroxyl radicals, generated primarily by NADPH oxidases and mitochondrial respiration (99). In immune cells, ROS serve not only as antimicrobial effectors but also as secondary messengers that shape innate immune signaling, regulating pathogen clearance, inflammatory activation, and cellular stress responses (100, 101). In this regard, RACK1 facilitates ROS production in immune cells for effective antimicrobial responses, while simultaneously mitigating excessive ROS accumulation in non-immune or stressed cells to prevent oxidative damage (102).

Mechanistically, RACK1 exerts this dual regulation by coordinating the activity and organization of the primary cellular ROS-generating systems. RACK1 was reported to be involved in NADPH oxidase complex assembly and mitochondrial homeostasis, which are the major sources of cellular ROS (31, 103). In macrophages, RACK1 facilitates the translocation of PKCβ to the plasma membrane, enabling phosphorylation of p47phox and subsequent assembly of the NADPH oxidase complex. This process promotes superoxide production and enhances the host’s antimicrobial capacity. Knockdown of RACK1 impairs this oxidative burst, leading to compromised innate immune responses. While PKCδ is also implicated in oxidative burst regulation in phagocytic cells, its specific role in RACK1-mediated NADPH oxidase activation remains to be further validated due to the non-specific off-target effects of its pharmacological inhibitors (104).

RACK1 has been shown to exert cell-protective effects by suppressing ROS accumulation under stress conditions. In hepatocellular carcinoma cells, RACK1 interacts with carbonyl reductase 1 (CBR1), a ubiquitous NADPH-dependent enzyme that protects cells from ROS-induced damage. This interaction stabilizes CBR1 by preventing its degradation and consequently reduces TNF-α-induced ROS accumulation, thereby promoting cell survival (105). Future research should elucidate the crosstalk between the RACK1/CBR1-ROS pathway and other TNF-α-induced signaling cascades, as targeting this axis may offer novel therapeutic strategies by modulating ROS production. In addition to directly inhibiting ROS accumulation, RACK1 also regulates ROS production by modulating autophagy-related processes (21). As an integral component of the 40S ribosomal subunit, RACK1 acts as a negative regulator of autophagy; its depletion triggers enhanced autophagic flux, which in turn affects mitochondrial turnover and ROS homeostasis (106). Depletion of ribosomal RACK1 alters the capacity of the ribosome to translate specific mRNAs, resulting in selective translation of mRNAs of genes for non-canonical autophagy induction (106). Additionally, RACK1 protects cells from oxidative stress via the DJ-1/AMPK/mTOR pathway induced adaptive autophagy. It has been reported that RACK1 interacts with DJ-1 to enhance AMPK activation and downstream mTOR signaling, conferring resistance to ROS-induced damage (107). These findings further elucidate the molecular mechanism by which RACK1 participates in hypoxia-induced autophagy and provide novel insights for the related disease pathologies.In summary, these findings illustrate the multifaceted role of RACK1 in ROS regulation. It promotes oxidative killing during innate immune activation while concurrently safeguarding cells from ROS overaccumulation under stress or pathological conditions. This dual role positions RACK1 as both a pro-inflammatory amplifier and a cellular stress modulator in the immune system.

### 3.5 Roles of RACK1 in adaptive immunity

Adaptive immunity is orchestrated by T and B lymphocytes, which arise from common hematopoietic progenitors (108). T cells mature in the thymus, where interactions between developing thymocytes and self-peptide–MHC complexes drive positive and negative selection, thereby shaping a diverse but self-tolerant T cell receptor repertoire (109). In contrast, B cells develop mainly in the bone marrow through a tightly regulated program involving immunoglobulin gene rearrangement, B cell receptor formation, and tolerance checkpoints, which together establish a diverse B cell receptor repertoire (110). Upon activation by antigen encounter in secondary lymphoid organs, T cells differentiate into effector and memory cells, while B cells become plasma cells that produce antibodies or long-lived memory B cells. This adaptability and memory function are key to protective immunity and underlie the success of vaccines and immunotherapies (111). T cells mediate cellular immunity by killing infected or abnormal cells and by supporting B cell maturation through helper T (Th) cell–driven signaling, such as Th1- and Th2-type responses (112). B cells, upon activation and interaction with Th cells, differentiate into plasma cells that secrete antigen-specific antibodies, forming the basis of humoral immunity (113). Both T and B cells generate memory populations that enable faster and stronger responses upon re-infection. In recent years, RACK1 has been identified as a critical regulator of lymphocyte development, activation, and differentiation, and its dysfunction leads to immune imbalance and increased disease susceptibility (43–45).

#### 3.5.1 RACK1 in T cell activation, differentiation, and homeostasis

T cell activation requires TCR engagement and co-stimulatory inputs that initiate cascades involving Src-family kinases such as Lck and downstream effectors like ZAP-70. Upon stimulation, RACK1 interacts with actinin-1 and Lck, forming a transient multiprotein complex that aids in immune synapse formation and TCR signaling efficiency (114, 115).

CD4^+^ T cell differentiation orchestrates both inflammatory and humoral responses during infection, with Th1 cells promoting pathogen clearance and T follicular helper (Tfh) cells supporting germinal center reactions and high-affinity antibody production (116). Th1 versus Tfh fate is determined by mutually exclusive expression of the lineage-defining transcription factors T-bet and Bcl6, whose activity is integrated by broader transcriptional networks (117). In this context, RACK1 regulates CD4^+^ T cell fate by modulating STAT3 stability. RACK1 deficiency impairs STAT3 activation and downregulates Bcl6, thereby limiting Tfh differentiation. Restoration of RACK1 or STAT3 in RACK1-deficient CD4^+^ T cells rescues STAT3 activity and Bcl6 induction, indicating that RACK1 promotes optimal STAT3 stability by inhibiting ubiquitin–proteasome–mediated degradation, thereby supporting Tfh cell development and germinal center responses (44).

RACK1 acts as an essential scaffold within the type I interferon (IFN) pathway. Usacheva et al. showed that RACK1 is recruited to the IFN receptor complex via IFNAR2 and is required for efficient STAT1/STAT2 activation and subsequent interferon-stimulated gene (ISG) transcription (63, 118). Consistent with this, genetic or pharmacologic disruption of RACK1 markedly blunts antiviral gene expression. Expanding beyond the canonical IFN-I axis, recent evidence positions RACK1 as a pleiotropic regulator that also modulates STAT3-dependent signaling cascades (44). Collectively, these observations implicate RACK1 as a point of convergence that couples IFN-mediated innate responses with STAT-dependent adaptive immune programs (44, 45). In T cells, RACK1 maintains cellular homeostasis and viability by regulating autophagy-dependent mitochondrial clearance; impaired clearance results in mitochondrial accumulation and compromised cell survival (45). T cell-specific deletion of RACK1 does not affect intrathymic development of conventional T cells or regulatory T cells (Tregs), but results in markedly reduced numbers of peripheral CD4^+^ and CD8^+^ T cells (45). These defects are cell-intrinsic, as demonstrated by impaired autophagy-dependent clearance of damaged mitochondria, increased accumulation of ROS, heightened susceptibility to apoptosis, and decreased proliferative capacity of peripheral T cells, indicating that RACK1 is essential for maintaining T cell homeostasis and survival (45).

Besides, RACK1 has been implicated in mediating “reverse signaling” downstream of the T cell co-stimulatory molecule CD40L. It interacts with signaling molecules such as PKC, JNK, and AKT, promoting T-cell proliferation and reducing apoptosis. Additionally, RACK1 facilitates STAT1 phosphorylation, stabilizing Th1 responses and sustaining IFN-γ production. In a mouse model, CD40L clustering was shown to engage RACK1 as a mediator of reverse signaling, stabilizing STAT1-associated pathways and enhancing IFN-γ production and Th1 polarization following CD40L engagement, although direct mechanistic validation of this pathway remains to be fully demonstrated (119). This study highlighted the critical role of RACK1 in reinforcing CD40L-CD40 forward signaling–driven immune responses. Future researches should explore the contribution of CD40L–RACK1 signaling to STAT1 phosphorylation and IFN−γ stability, which may reveal a distinct layer of regulation in Th1 polarization.

Recent studies have further delineated RACK1 as a critical regulator of adaptive immunity in the context of infection and immune dysregulation. In models of parasitic challenge, RACK1 is required for optimal Tfh cell development and function (44). Its action enhances germinal center formation and high-affinity antibody generation, thereby promoting effective humoral responses following *Plasmodium* infection. Beyond canonical helper T cell differentiation, single-cell profiling of immune microenvironments in Mtb/HIV co-infection has identified a RACK1-expressing CD8^+^ T cell subset characterized by high TIGIT expression, the targeting of which can reinvigorate effector capacity and may ameliorate pathogen persistence, underscoring RACK1’s role in shaping cytotoxic T cell responses under chronic infectious stress (120). Besides, RACK1 also intersects with immune regulation in cancer models. The deubiquitinase USP17LA interacts with and stabilizes RACK1 by antagonizing its ubiquitin-dependent degradation, thereby facilitating RACK1-mediated NFAT suppression and subsequent attenuation of effector T cell function, with implications for anti-tumor immunity (121). In murine models of T cell–mediated autoimmune and inflammatory disorders, loss of the acetyltransferase NAT10 leads to decreased peripheral T cell numbers. Mechanistically, NAT10 acetylates RACK1 at K185 to prevent its K48-linked ubiquitination and degradation. Enhanced RACK1 stability in this setting reprograms ribosome biogenesis and metabolic flux to support T cell proliferation, illustrating how post-translational modulation of RACK1 integrates cellular metabolism with immune effector states (122).

Notably, emerging evidence indicates that RACK1 also regulates immune homeostasis by shaping Treg function through noncanonical inflammasome-related mechanisms. In Tregs, RACK1 partners with AIM2 to control cellular metabolism and reinforce the suppressive phenotype, independently of classical inflammasome activation (123). Although this pathway was initially characterized in autoimmune settings, including experimental models of multiple sclerosis and inflammatory bowel disease, its implications extend beyond autoimmunity (123). During infection, effective host defense requires a finely tuned balance between robust effector responses and the timely engagement of regulatory programs to prevent excessive immunopathology (124). In this context, the RACK1-AIM2 axis may serve as a molecular switch that restrains overactive inflammation by stabilizing Treg metabolic fitness and suppressive capacity once pathogen control is initiated (125, 126). Thus, RACK1-dependent regulation of Treg function provides a conceptual framework linking immune tolerance mechanisms with infection-induced immune responses, underscoring RACK1’s dual role in coordinating immune activation and resolution to maintain tissue integrity and host survival.

#### 3.5.2 RACK1 in B cell development and effector functions

B cell development occurs through a tightly regulated differentiation program in the bone marrow, integrating immunoglobulin gene rearrangement, receptor-mediated selection, and survival signaling to establish a functional and self-tolerant repertoire. Mature B cells subsequently diversify into distinct subsets with specialized effector capacities that underpin humoral immune responses (110). In this regard, Pax5 is a master transcription factor required for B cell lineage commitment, immunoglobulin gene rearrangement, and the repression of alternative hematopoietic lineage programs (127). It enforces B cell identity by activating B cell–specific genes, including Cd19 and mb-1, while silencing genes associated with myeloid or T cell fates, such as Notch1, Csf1r, and Flt3, and by regulating accessibility of immunoglobulin heavy-chain *V_H* segments during BCR assembly (43, 128). Recent work has identified RACK1 as a critical post-translational regulator of Pax5 stability. RACK1 directly binds Pax5 and inhibits its ubiquitination and proteasomal degradation (43). Accordingly, deletion of RACK1 in early B cells leads to Pax5 destabilization, resulting in a developmental arrest at the pro-B cell stage and a marked reduction in transitional and follicular B cell populations. Notably, ectopic expression of Pax5 partially rescues B cell development in RACK1-deficient pro-B cells, establishing RACK1-Pax5 interaction as a key mechanism sustaining B lymphopoiesis and B cell identity (43).

Beyond its role in maintaining lineage commitment, RACK1 also contributes to signaling competence in later stages of B cell differentiation. Conditional deletion of RACK1 in the B cell lineage demonstrates that RACK1 is essential for normal B cell development and signaling (43, 52). Loss of RACK1 blocks B cell progression at the pre-B cell stage and impairs V(D)J recombination, defects that are not rescued by enforced Bcl2 expression. In mature B cells, RACK1 supports BCR-dependent MAPK and NF-κB signaling, thereby enabling effective proliferation, class-switch recombination, and antigen-specific antibody production (52). Additionally, RACK1 has recently been reported to regulate mucosal immunity by coordinating the early activation of B cells, thereby modulating AKT phosphorylation and subsequent differentiation of mucosal B cells. Chang et al. identified an Fgl2-Rack1 axis that restrains B cell mucosal immunity by limiting Rack1-mediated AKT phosphorylation. Fgl2 deficiency expands marginal zone B cells, germinal center B cells, and IgA^+^ plasma cells, effects reversed by RACK1 inhibition *in vivo* and *in vitro* (129). These findings position RACK1 as a potential therapeutic target for modulating humoral immunity.

## 4 Roles of RACK1 in different pathogen infections

### 4.1 RACK1-mediated mechanisms during bacterial infections

RACK1 plays a dual role in bacterial infection. It functions as a key component of host antibacterial signaling, yet its scaffolding function also makes it vulnerable to exploitation by diverse bacterial effectors (42, 84). This reflects an evolutionary pattern in host-pathogen interactions, where signaling nodes can coordinate immune responses but also serve as targets for pathogens (130, 131). Accordingly, recent studies reveal context-specific outcomes, in which the impact of RACK1 engagement depends on the pathogen’s effector repertoire, the infected cell type, and the temporal progression of infection (42). In this section, we summarize the roles of RACK1 in different bacterial infections (**Table 1**).

**Table 1:** Roles of RACK1 in bacterial infections.

Bacteria	Cell lines/model	Effect	Mechanism	Consequence	Reference
Mycobacterium tuberculosis (*M. tuberculosis*)	Macrophages, intracellular M. tuberculosis infection model	Promotes host defense	EST12 binds RACK1 WD5-7, recruits UCHL5, deubiquitinates NLRP3	IL-1β/GSDMD activation, IL-6/TNF-α/iNOS induction, M1 polarization, reduced bacterial burden.	(40, 132)
Streptococcus suis (*S. suis*)	Porcine macrophages, inflammasome activation model		RACK1 interacts with NEK7 and facilitates NLRP3-NEK7 complex assembly.	More GSDMD cleavage and IL-1β secretion in infected porcine macrophages.	(97)
*Pasteurella multocida* (*P. multocida*)	Macrophages, inflammatory infection model		RACK1 enhances NLRP3 activation and inducible NF-κB signaling.	Higher p65 phosphorylation and CXCL1/CXCL2/IL-6/IL-12/TNF-α, reduced adhesion/invasion, better clearance.	(41)
*Shigella flexneri* (*S. flexneri*)	*C. elegans*, host-defense infection model		RACK-1 acts upstream of or together with conserved MAPK signaling.	Stronger antimicrobial responses and greater resistance to infection.	(82)
Klebsiella pneumoniae (*K. pneumoniae*)	*C. elegans*, probiotic-modulated infection model		*L. casei* activates RACK-1 through a TLR-p38 MAPK pathway.	Higher antimicrobial gene expression and improved host defense.	(86)
Helicobacter pylori (*H. pylori*)	Gastric epithelial cells, *H. pylori* infection model	Loss of RACK1 promotes bacterial infection	Infection suppresses RACK1, increasing integrin β1-dependent NF-κB signaling.	Greater adhesion/colonization, chronic inflammation, and tumor-prone progression.	(68)
Yersinia pseudotuberculosis (*Y. pseudotuberculosis*)	Host phagocytic cells, phagocytosis/evasion model	Bacterial effector antagonizes RACK1	YopK binds RACK1 WD3-4 and blocks the RACK1-FAK interaction.	Inhibits phagocytic cup formation and supports extracellular survival.	(133)

RACK1, receptor for activated C kinase 1; M90T, Shigella flexneri M90T strain; EST12, ESAT-6 secretion system 12 kDa protein; WD5-7, WD repeat domains 5–7; UCHL5, ubiquitin carboxyl-terminal hydrolase L5; NLRP3, NLR family pyrin domain containing 3; NEK7, NIMA-related kinase 7; MAPK, mitogen-activated protein kinase; TLR-p38, Toll-like receptor-p38 mitogen-activated protein kinase; RACK1-FAK, receptor for activated C kinase 1-focal adhesion kinase; IL-1, interleukin-1; GSDMD, gasdermin D; IL-6, interleukin-6; TNF-α, tumor necrosis factor-alpha; iNOS, inducible nitric oxide synthase; CXCL1/CXCL2, C-X-C motif chemokine ligand 1/C-X-C motif chemokine ligand 2.

#### 4.1.1 RACK1 as an antibacterial signal integrator

Accumulating evidence supports a role for RACK1 as a scaffolding platform that coordinates innate immune signaling downstream of PRRs during bacterial infection (42). In the context of *M. tuberculosis*, the secreted effector EST12 directly interacts with the WD5–7 repeats of RACK1, thereby facilitating the recruitment of the deubiquitinase UCHL5 (40). This complex promotes the removal of K48-linked ubiquitin chains from NLRP3, relieving its basal repression and enabling caspase-1 activation and downstream inflammasome signaling (40). Notably, this function appears to be selective, as RACK1 preferentially supports NLRP3 activation in response to specific bacterial cues rather than acting as a universal inflammasome scaffold (31).

Beyond immediate inflammasome activation, RACK1 has been implicated in shaping delayed transcriptional responses during bacterial infection. In *M. tuberculosis*–infected macrophages, the EST12-RACK1 complex activates the JNK pathway, leading to phosphorylation of c-Jun and c-Fos, formation of AP-1 complexes, and subsequent induction of Myc expression (132). Myc in turn promotes transcription of antimicrobial effector genes, including IL-6, TNF-α, and iNOS, and supports polarization toward an M1-like macrophage phenotype (132, 134).

A mechanistically distinct mode of action has been described in *Streptococcus suis* infection. In this setting, RACK1 interacts with NEK7 and promotes the formation of the NLRP3-NEK7 complex, thereby facilitating inflammasome assembly. Post-translational modification of RACK1, including tyrosine phosphorylation, may contribute to this process, although the precise upstream kinases and specific residues involved remain to be fully defined (27, 97). RACK1 depletion significantly reduces GSDMD cleavage and IL-1β secretion in infected porcine macrophages, indicating that RACK1 contributes to efficient inflammasome signaling (97). However, inflammasome readouts were attenuated rather than abolished upon RACK1 knockdown, suggesting that RACK1 acts as a facilitating or rate-limiting factor rather than an indispensable structural component (97).

In *P. multocida* infection, RACK1 expression is reduced during infection, whereas experimental overexpression enhances NLRP3 activation and bacterial clearance (42). In addition, RACK1 promotes NF-κB activation, as reflected by increased p65 phosphorylation and elevated expression of NF-κB-dependent cytokines and chemokines during *P. multocida* infection (41, 42). This is associated with enhanced NF-κB activation, reflected by increased p65 phosphorylation and elevated transcription of NF-κB–dependent cytokines and chemokines, including CXCL1, CXCL2, IL-6, IL-12, and TNF-α, and correlates with reduced bacterial adhesion and invasion (42). In macrophages, RACK1 therefore functions as a positive regulator that amplifies inducible NF-κB signaling without substantially altering basal pathway activity.

These observations raise the possibility that RACK1 may operate in distinct subcellular pools with separable functions, potentially balancing roles in bacterial interaction at the plasma membrane and inflammasome licensing in the cytosol. At present, direct evidence supporting this spatial segregation model is lacking (27).

In contrast, in *H. pylori*-infected gastric epithelial cells, RACK1 expression is reduced, and RACK1 depletion paradoxically enhances NF-κB activation through increased integrin β1 signaling, exacerbating chronic inflammation and promoting carcinogenic progression (68). These opposing outcomes underscore the cell-type- and receptor-specific nature of RACK1-mediated NF-κB regulation. In macrophages, RACK1 primarily amplifies infection-induced NF-κB outputs in macrophages, whereas in epithelial cells it appears to restrain integrin-dependent inflammatory signaling (42, 135).

#### 4.1.2 Bacterial hijacking of RACK1: from invasion to immune evasion

Pathogens have evolved to turn RACK1’s scaffolding property to their advantage (136). *S. flexneri* effectors IpaB and IpaC activate host Src kinase, which phosphorylates RACK1 at Tyr−228, creating a binding site for the WAVE regulatory complex (84, 137). The resulting RACK1−WAVE assembly accelerates Arp2/3−mediated actin polymerization, generating membrane ruffles that engulf bacteria (84). In human intestinal organoids, RACK1 knockdown significantly reduces *Shigella* invasion and limits intercellular spread (84), confirming its proviral role. Critically, RACK1 does not directly contact actin; it is a signaling coordinator that recruits the actin nucleation machinery to the point of entry.

*Yersinia pseudotuberculosis* employs the opposite tactic: its type−III effector YopK binds RACK1’s WD3–4 repeats, preventing RACK1 from interacting with focal adhesion kinase (FAK) and thereby blocking phagocytic cup formation (84, 138). By sequestering RACK1, *Yersinia pseudotuberculosis* disables the host’s uptake machinery, ensuring its extracellular survival. The molecular interface of YopK−RACK1 has not been mapped, and it is unknown whether YopK competes with other RACK1 ligands (e.g., PKC) for binding (133).

### 4.2 RACK1-mediated mechanisms during viral infections

**Figure 5:**
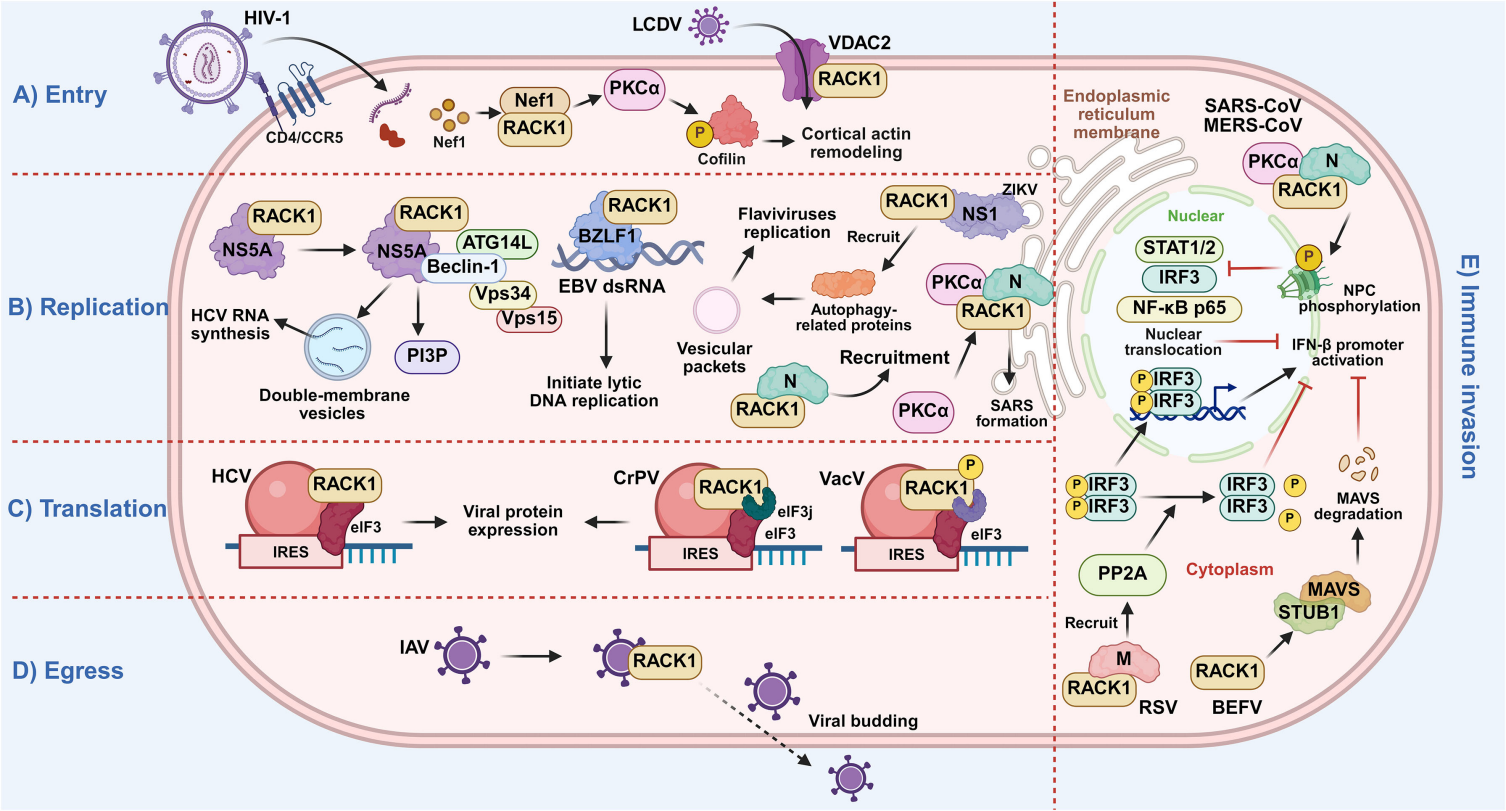
RACK1-mediated mechanisms during viral infections. **(A)** Entry: RACK1 associates with VDAC2 to facilitate LCDV viral entry into host cells. RACK1 binds to the Nef1 protein, recruits PKCα to achieve cofilin phosphorylation, and promotes cortical actin remodeling. RACK1 facilitates HIV-1 entry through cytoplasmic scaffolding functions. **(B)** Replication: For HCV, RACK1 interacts with NS5A to assemble a complex that promotes PI3P production and double-membrane vesicle formation for RNA synthesis. In ZIKV and SARS-CoV-2, RACK1 recruits autophagy factors or PKCα to remodel membranes for replication organelle biogenesis. In EBV, RACK1 stabilizes BZLF1 in the nucleus to drive lytic replication. **(C)** Translation: RACK1 interacts with eIF3 subunits to modulate IRES-dependent translation of HCV and CrPV viral RNAs. **(D)** Egress: RACK1 interacts with IAV particles to facilitate viral release and dissemination. **(E)** Immune invasion: SARS-CoV and MERS-CoV use the N protein to form a complex with RACK1 and p-PKCα, leading to NPC phosphorylation and redistribution. This blocks nuclear translocation of key transcription factors, suppressing IFN-β production. In RSV, RACK1 and the M protein recruits PP2A, which dephosphorylates IRF3, preventing its activation and IFN-β induction. In BEFV, RACK1 promotes the degradation of MAVS, thereby inhibiting IFN-β promoter activation. ATG14L, autophagy-related 14-like protein; BZLF1, BamHI Z leftward frame 1; CD4, cluster of differentiation 4; CCR5, C-C chemokine receptor 5; CrPV, cricket paralysis virus; dsRNA, double-stranded RNA; EBV, Epstein-Barr virus; eIF3, eukaryotic initiation factor 3; HCV, hepatitis C virus; HIV-1, human immunodeficiency virus type 1; IAV, influenza A virus; IFN-β, interferon-beta; IRES, internal ribosome entry site; IRF3, interferon regulatory factor 3; LCDV, lymphocystis disease virus; M, matrix protein; MERS-CoV, Middle East respiratory syndrome coronavirus; N, nucleoprotein (nucleocapsid protein); Nef1, negative factor 1; NF-κB, nuclear factor kappa B; NPC, nuclear pore complex; NS1, non-structural protein 1; NS5A, non-structural protein 5A; p65, NF-κB subunit p65 (RelA); PI3P, phosphatidylinositol 3-phosphate; PKCα, protein kinase C alpha; PP2A, protein phosphatase 2A; RACK1, receptor for activated C kinase 1; RSV, respiratory syncytial virus; SARS-CoV, severe acute respiratory syndrome coronavirus; STAT1/2, signal transducer and activator of transcription 1/2; VACV, vaccinia virus; VDAC2, voltage-dependent anion channel 2; Vps15, vacuolar protein sorting 15; Vps34, vacuolar protein sorting 34; STUN1, U-box containing protein 1; MAVS, mitochondrial antiviral signaling protein; BEFV, bovine ephemeral fever virus; ZIKV, Zika virus.

Beyond its established roles in bacterial infection, RACK1 functions as a key host factor for a range of viruses, coordinating multiple stages of the viral life cycle while also modulating innate immune responses (139) (**Table 2**). As a seven-bladed β-propeller scaffold, RACK1 provides multiple protein-interaction surfaces that allow it to assemble diverse signaling and ribosome-associated complexes (46). In viral infection, this scaffolding capacity can be used either by the host to regulate antiviral signaling or by viruses to support replication and immune evasion, depending on the viral species and cellular context (**Figure 5**).

**Table 2:** Roles of RACK1 in viral infections.

Virus	Family	Genome	Stage	Mechanism	Consequence	Reference
Lymphocystis disease virus (LCDV)	Iridoviridae	dsDNA	Entry	RACK1 forms a VDAC2 receptor complex and supports caveolae-mediated endocytosis.	Antibody or siRNA blockade markedly reduces viral entry.	(140)
Human immunodeficiency virus type 1 (HIV-1)	Retroviridae	ssRNA-RT	Entry	Nef binds RACK1 and recruits PKCα to phosphorylate cofilin and remodel cortical actin.	More efficient virological synapse transmission between T cells.	(141)
Pseudorabies virus (PRV)	Orthoherpesviridae	dsDNA	Replication	RACK1 enhances STING-dependent IRF3 phosphorylation and type I IFN activation.	Depletion increases replication, whereas overexpression reduces viral production.	(63)
Hepatitis C virus (HCV)	Flaviviridae	(+)ssRNA	Replication	NS5A engages RACK1 and assembles the NS5A-RACK1-ATG14L-Beclin1-Vps34-Vps15 complex.	Promotes PI3P production, DMV biogenesis, and viral RNA synthesis.	(142, 143)
Zika virus (ZIKV)				RACK1 interacts with NS1 at the ER membrane and recruits autophagy-related proteins.	Supports vesicular packet and replication-organelle formation.	(139)
Dengue virus (DENV)				The conserved RACK1-NS1 axis maintains ER membrane curvature and replication compartments.	RACK1 depletion abolishes viral RNA replication.	(144)
Severe acute respiratory syndrome coronavirus 2 (SARS-CoV-2)	Coronaviridae		Replication	N protein binds RACK1 and recruits PKCα at the ER membrane.	Drives membrane remodeling and DMV formation required for replication.	(145)
Epstein-Barr virus (EBV)	Orthoherpesviridae	dsDNA	Replication	RACK1 binds BZLF1/ZEBRA and stabilizes the latent-to-lytic switch machinery.	Enhances lytic DNA replication.	(146, 147)
Cricket paralysis virus (CrPV)	Dicistroviridae	(+)ssRNA	Translation	RACK1 is required for 5’UTR IRES but dispensable for IGR IRES.	Supports nonstructural protein synthesis but not capsid IGR-driven translation.	(148, 149)
Vaccinia virus (VacV)	Poxviridae	dsDNA	Translation	B1R kinase phosphorylates RACK1 Ser278 to enhance late mRNA translation; RACK1 also supports MAVS aggregate formation.	Boosts viral gene expression but can also enhance type I IFN signaling in other settings.	(150, 151)
Herpes simplex virus 1 (HSV-1)	Orthoherpesviridae		Translation	Infection-associated RACK1 phosphorylation favors IRES-dependent viral translation.	Dependency on RACK1 remains partly controversial across models.	(152, 153)
Influenza A virus (IAV)	Orthomyxoviridae	(−)ssRNA, segmented	Egress	M1 Pro16 binds RACK1 WD5-7 and stabilizes M1 at the plasma membrane.	Supports efficient budding and infectious virion release.	(154)

LCDV, Lymphocystis disease virus; RACK1, receptor for activated C kinase 1; VDAC2, voltage-dependent anion channel 2; siRNA, small interfering RNA; HIV-1, human immunodeficiency virus type 1; Nef, negative factor, PKCα, protein kinase C alpha; PRV, pseudorabies virus; STING, stimulator of interferon genes; IRF3, interferon regulatory factor 3; IFN, interferon; HCV, hepatitis C virus; NS5A, nonstructural protein 5A; ATG14L, autophagy-related 14-like protein; Beclin1, Beclin 1; Vps34, vacuolar protein sorting 34; Vps15, vacuolar protein sorting 15; PI3P, phosphatidylinositol 3-phosphate; DMV, double-membrane vesicle; ZIKV, Zika virus; NS1, nonstructural protein 1; ER, endoplasmic reticulum; DENV, dengue virus, SARS-CoV-2, severe acute respiratory syndrome coronavirus 2; EBV, Epstein-Barr virus; BZLF1, BamHI Z fragment leftward open reading frame 1; ZEBRA, Z Epstein-Barr replication activator; CrPV, Cricket paralysis virus; UTR, untranslated region; IRES, internal ribosome entry site; IGR, intergenic region; VACV, Vaccinia virus; B1R, vaccinia virus B1 kinase; MAVS, mitochondrial antiviral-signaling protein; HSV-1, herpes simplex virus 1; IAV, influenza A virus; M1, matrix protein 1; Pro16, proline at position 16, WD5-7, WD repeat domains 5-7.

#### 4.2.1 Entry

Productive infection requires viral attachment and entry into target cells. Most viruses enter cells through endocytic pathways, including clathrin-mediated endocytosis, macropinocytosis, and caveolae-mediated uptake, while some enveloped viruses can also enter by direct fusion with the plasma membrane (155, 156). Host scaffold proteins such as RACK1 can facilitate viral entry by organizing receptor complexes at the cell surface (142). In the case of lymphocystis disease virus (LCDV), a member of the Iridoviridae family that infects fish, RACK1 forms a functional receptor complex with voltage-dependent anion channel 2 (VDAC2) on the plasma membrane of flounder gill cells. This complex mediates caveolae-dependent endocytosis, as shown by receptor blockade experiments in which specific antibodies or small interfering RNAs markedly reduce viral entry (140, 157). In lymphocystis disease virus (LCDV) infection, VDAC2 and RACK1 have been identified as functional receptors that mediate viral entry into flounder gill cells. Subsequent work showed that LCDV enters these cells through a caveolae-mediated endocytic pathway facilitated by VDAC2 and RACK1, relying on dynamin and microtubules in a pH-independent manner (140, 158).

In contrast to its role as a plasma membrane receptor, RACK1 facilitates HIV-1 entry through cytoplasmic scaffolding functions. The HIV-1 Nef accessory protein directly binds RACK1 via its N-terminal region, recruiting PKCα to phosphorylate cofilin and modulate actin dynamics. This RACK1-PKCα axis enhances cortical actin remodeling at the virological synapse, promoting efficient viral transmission between T cells (141). While RACK1 does not serve as a primary receptor for HIV-1, this mechanism illustrates how RACK1 can be co-opted to optimize entry efficiency in susceptible cells.

#### 4.2.2 Replication

Following entry, viral genomes exploit host machineries for replication. RACK1 coordinates this process for diverse viruses (139, 144). Notably, RACK1 does not always promote viral replication. In pseudorabies virus infection, RACK1 acts as a restriction factor. Its depletion increases viral replication, whereas overexpression reduces viral production. This effect is associated with enhanced STING-dependent IRF3 phosphorylation and type I interferon activation (63). For hepatitis C virus (HCV), the nonstructural protein 5A (NS5A) directly engages RACK1 through its D1 domain (amino acids 31–213) (142). This interaction nucleates the assembly of the NS5A-RACK1-ATG14L-Beclin1-Vps34-Vps15 complex (142). This complex supports PI3P production and promotes the biogenesis of double-membrane vesicles (DMVs) that serve as dedicated sites for viral RNA synthesis (143).

RACK1’s scaffolding function in replication organelle biogenesis extends to flaviviruses and coronaviruses through analogous mechanisms, although the precise molecular interfaces and recruited host factors vary across viral families. In Zika virus (ZIKV)-infected cells, RACK1 interacts with the viral NS1 protein at the endoplasmic reticulum (ER) membrane, recruiting autophagy-related proteins to facilitate the formation of vesicular packets—the characteristic replication organelles of flaviviruses (139).

In dengue virus (DENV) infection, RACK1 functions as a proviral host factor within the viral replication complex. Mechanistically, RACK1 acts as a scaffold platform on the 40S ribosomal subunit, recruiting Vigilin and SERBP1. These RNA-binding host-dependency factors interact with both RACK1 and the DENV viral RNA, serving as molecular linkers that connect the viral genome to the host translation machinery, thereby facilitating the essential viral translation and replication steps during infection (144).

For SARS-CoV-2, the nucleocapsid (N) protein binds RACK1 to promote PKCα recruitment to the ER membrane. This RACK1-N-PKCα complex orchestrates membrane remodeling events required for DMV formation, with pharmacological inhibition of PKCα or RACK1 knockdown significantly reducing viral replication (145).

In Epstein-Barr virus (EBV), the immediate-early protein BZLF1, also known as ZEBRA or Zta, functions as a lytic switch factor that regulates the transition from latency to lytic replication (146). RACK1 has been identified as a cellular binding partner of BZLF1. In an early study, yeast two-hybrid and co-immunoprecipitation assays showed that RACK1 interacts with the amino-terminal transactivation domain of BZLF1, suggesting that RACK1 may modulate BZLF1-associated regulatory complexes rather than acting as a general mediator of EBV lytic replication (147). This nuclear scaffolding function highlights RACK1’s versatility in supporting both cytoplasmic and nuclear phases of viral replication.

#### 4.2.3 Translation

RACK1 modulates eukaryotic translation through both free and ribosome-associated pools. Ribosome-bound RACK1, positioned at the mRNA exit channel of the 40S subunit, functions as a key translational regulator (159). RACK1 exerts profound control over viral protein synthesis, particularly for viruses utilizing internal ribosome entry site (IRES)-dependent translation. The HCV IRES represents the paradigm for RACK1-mediated translational regulation. In Huh7.5.1 cells, RACK1 depletion selectively impairs IRES-driven translation of the HCV polyprotein without affecting global cap-dependent translation, leading to a marked reduction in viral protein expression (160, 161).

Mechanistically, RACK1 bridges the IRES to the eIF3j subunit, facilitating the assembly of 48S preinitiation complexes on the viral RNA (161). This function extends to the dicistrovirus cricket paralysis virus (CrPV) (148), where RACK1 is specifically required for 5’UTR IRES-mediated translation of nonstructural proteins but dispensable for intergenic region (IGR) IRES-driven capsid protein synthesis (149). The differential requirement reflects the unique ability of IGR IRESs to initiate translation without initiation factors, whereas 5’UTR IRESs rely on RACK1-eIF3j scaffolding (149).

Notably, RACK1-dependent translation is also subject to viral subversion through post-translational modification. Park et al. found that during vaccinia virus (VacV) infection, JNK activation is indirectly attenuated due to reduced accumulation of viral proteins, whereas RACK1 is essential for JNK phosphorylation induced by ribosomal toxicity stress. The viral B1 kinase phosphorylates RACK1 at serine 278, a modification that promotes efficient translation of post-replicative viral mRNAs bearing 5′ poly(A) leaders (150). Their recent work further showed that, during VacV-induced host shutoff, translation of viral mRNAs depends on RACK1 and eIF3, whereas selected host transcripts such as *JUN* use a distinct initiation strategy. Cryo-electron microscopy analysis revealed that eIF3-bound 40S ribosomes from VacV-infected cells exhibit an expanded rotational range of the RACK1-containing 40S head domain, indicating infection-induced remodeling of the translation initiation machinery. This remodeling enables the differential RACK1/eIF3 dependencies, allowing distinct initiation strategies for viral and select host mRNAs to operate concurrently during shutoff (151).

Collectively, these studies establish RACK1 as a critical host dependency factor for the translation of viruses that rely on IRES-dependent or non-canonical initiation mechanisms (153, 155). However, this dependency is not universal: genetic ablation of RACK1 does not impair herpes simplex virus 1 (HSV-1) translation, as HSV-1 encodes its own ribosome-associated factors to bypass host RACK1 (152). These pathogen-specific dependencies suggest that selectively targeting virus-RACK1 translation interfaces, rather than indiscriminate RACK1 suppression, may offer a more viable antiviral strategy. Thus, dissecting virus-specific RACK1 dependencies and developing selective inhibitors that disrupt pathogen-engaged RACK1 complexes may yield targeted antiviral therapeutics against RACK1-dependent viruses.

#### 4.2.4 Egress

Viral egress, primarily via cell lysis or budding, follows maturation in infected cells. RACK1’s role in this process remains an emerging research focus (136). The IAV matrix protein M1 contains a proline-rich motif at position 16 that directly interacts with RACK1’s WD5–7 repeats. This interaction stabilizes M1 at the plasma membrane and facilitates the budding of viral particles, as demonstrated by RACK1 knockdown studies showing a 60% reduction in infectious virion release (154). Mutation of the proline residue abrogates M1-RACK1 binding and impairs viral egress without affecting intracellular viral RNA replication, establishing a specific role for RACK1 in the final assembly step (162).

#### 4.2.5 Immune evasion

Beyond its direct roles in the viral life cycle, RACK1 is extensively co-opted by viruses to dismantle host innate immune responses, particularly IFN signaling. Coronaviruses employ a sophisticated immune evasion strategy centered on RACK1-mediated disruption of nucleocytoplasmic trafficking. The SARS-CoV-2 N protein forms a ternary complex with RACK1 and activated PKCα (p-PKCα) in the cytoplasm, which phosphorylates nuclear pore complex (NPC) protein NUP62 at serine 243 (145). This phosphorylation triggers NUP62 redistribution from the NPC to the cytoplasm, creating a physical barrier that blocks nuclear translocation of STAT1, STAT2, IRF3, and NF-κB p65 (145, 161). Consequently, IFN-β transcription and downstream antiviral gene expression are profoundly suppressed. This mechanism is conserved across beta-coronaviruses, with N proteins from SARS-CoV and MERS-CoV demonstrating comparable RACK1-dependent immune modulation (145).

Respiratory syncytial virus (RSV) adopts an alternative RACK1-dependent evasion strategy that operates at the level of IRF3 activation. The RSV matrix (M) protein interacts with RACK1 to recruit protein phosphatase 2A (PP2A), which dephosphorylates IRF3 at serine 396. This dephosphorylation prevents IRF3 dimerization and nuclear accumulation, thereby silencing IFN-β promoter activation (163, 164). Disruption of the M–RACK1–PP2A axis through RACK1 knockdown or PP2A inhibitors restores IRF3 activation and suppresses RSV replication, highlighting this pathway as a viable therapeutic target (163, 164).

Bovine ephemeral fever virus (BEFV) targets the same innate immune axis through a mechanistically distinct route. BEFV upregulates RACK1 expression to promote E3 ligase STUB1-mediated degradation of MAVS, thereby abrogating upstream RIG-I/MAVS signaling and suppressing IFN production (165).

These divergent mechanisms illustrate how distinct viral families have evolved specialized strategies to repurpose RACK1 for immune evasion at distinct nodes of the IFN signaling cascade, underscoring the context-dependent roles of RACK1 in viral pathogenesis.

### 4.3 Therapeutic implications

RACK1 plays a unique role in the interactions among host translational control and innate immunity, making it an attractive but inherently risky therapeutic target (166). Early pharmacological efforts focused predominantly on oncology, exploiting strategies that disrupt protein–protein interactions (PPIs), functionally uncouple signaling complexes, or degrade the scaffold through proteolysis-targeting chimera (PROTAC) technology (46). More recently, this mechanistic repertoire has been extended to infectious disease, where RACK1’s role as a physical platform for viral replication complexes presents a compelling rationale for host-directed antiviral intervention.

The antiviral strategy hinges on the structural plasticity of RACK1’s seven-bladed β-propeller. Structure-based inhibitor design enables spatially selective engagement. SD-29 and harringtonolide (HA) occupy distinct WD-repeat blades, competitively occupying core binding sites or inducing conformational changes to block specific PPIs while potentially sparing essential ribosome-associated complexes (167, 168). A recent chemo-proteomic screen identified SB2960, a benzopyranyl-pyrazole that binds RACK1 and allosterically remodels stress granule dynamics to enhance antiviral gene expression, suppressing replication across multiple viruses including SARS-CoV-2 with low cytotoxicity and marked synergy with remdesivir (169). For a catalytically inactive scaffold, elimination strategies offer an alternative to occupancy-driven inhibition. RNA interference and PROTACs can remove the entire RACK1 platform, abrogating scaffolding functions that conventional inhibitors cannot touch (170, 171). Ultimately, exploiting infection-induced post-translational modifications to generate disease-specific RACK1 conformers may enable allosteric modulators or conditional degraders that act selectively during infection progression without compromising housekeeping ribosomal functions (172, 173).

Despite these advances, these approaches remain limited by delivery efficiency, off-target ubiquitination, and lack of optimized *in vivo* delivery systems. No direct agonist of RACK1 is available, and all activation strategies rely on indirect modulation, while existing inhibitors such as SB2960 remain at the proof-of-concept stage without co-crystal structural validation or preclinical advancement for bacterial infections (46). Moreover, translation from cellular models to clinical application remains constrained by substantial technological and biological gaps (174, 175). Targeting RACK1 for therapeutic purposes is complicated by its nature as a multifunctional scaffold protein. Although RACK1 depletion appears minimally cytotoxic in certain cell lines, this observation does not fully recapitulate the systemic risks of pharmacological suppression. The protein’s integration into the 40S ribosomal subunit and its scaffolding of essential signaling kinases raise concerns about specificity, off-target effects, and the disruption of normal cellular homeostasis (27). Moreover, RACK1 suppression poses inherent risks to protein synthesis homeostasis and long-term viability in highly proliferative tissues, given its indispensable role in ribosome function (29).These limitations highlight the issue of broad RACK1 modulation and demand the development of strategies that achieve pathway-specific modulation while sparing essential housekeeping functions (27).

Although the therapeutic implications of RACK1 have been gradually identified (168, 176), several substantive issues remain incompletely resolved. First, the structural determinants of RACK1 selectivity need to be elucidated at atomic resolution, defining interaction interfaces that are unique to viral infection contexts and thus druggable without compromising ribosomal integrity (48). Second, the post-translational modification landscape of RACK1 during infection—phosphorylation, acetylation, and ubiquitination events that create “disease-specific” conformers—must be mapped with temporal precision to enable the design of conditionally active modulators (150). Third, single-cell transcriptomic analyses of infected tissues are needed to dissect cell-to-cell heterogeneity in viral replication and innate immune signaling, identifying which cellular subpopulations would benefit from RACK1 modulation and which would be most vulnerable to its loss (177). Integrating chemical proteomics, structural biology, and single-cell systems immunology will be essential to translate RACK1’s central hub status into the next generation of precision anti-infective therapies (27).

## 5 Conclusion and future perspectives

RACK1 is increasingly recognized as an important regulator that links immune signaling with translational control during host responses to infection. Across innate and adaptive immunity, RACK1 acts not as an isolated signaling component but as a scaffold that coordinates inflammatory signaling, inflammasome activity, redox regulation, and lymphocyte differentiation. The effects of RACK1 depend strongly on cellular context, allowing it to either support antimicrobial defense or be exploited by pathogens during infection. This dual role reflects its involvement in both signaling pathways and protein synthesis processes. Therefore, targeted therapeutic strategies must center on the bidirectional regulatory function of RACK1 and integrate its distinct pathway-specific regulatory characteristics. Therapeutically, RACK1 represents a promising but challenging target owing to its multifunctional scaffold nature and ribosomal integration. Pathway-selective modulation offers a viable route to disrupt pathogen-exploited interactions while preserving essential functions. Integrating these therapeutic insights with mechanistic understanding will be essential for developing precise host-directed therapies against bacterial and viral infections.

Currently, treatment strategies targeting RACK1 still face substantial biological and pharmacological challenges. First, the structural basis that determines how RACK1 selectively interacts with host and pathogen partners is still unclear, and the regulation of different ribosomal and signaling-associated RACK1 pools remains poorly understood. Second, as a multifunctional scaffold protein, RACK1 presents significant pharmacological challenges, including poor target specificity, off-target effects, and the difficulty of achieving pathway-specific modulation without interfering with physiological processes. Current pharmacological approaches still lack the refinement to selectively disrupt pathological RACK1 complexes while sparing homeostatic ones. Third, the potential toxicity associated with broadly targeting a protein essential for both immune signaling and protein synthesis necessitates the development of strategies to mitigate adverse effects. Therefore, realistic therapeutic prospects must explicitly acknowledge these current technological limitations, and future studies combining structural biology, single-cell analysis, and advanced pharmacological platforms will be essential to establish a balanced translational perspective for RACK1-directed therapies.
